# Identification of clinically relevant multi-drug resistant ESKAPEE isolates from hospital wastewater surveillance in Thailand

**DOI:** 10.3389/fmicb.2025.1657219

**Published:** 2025-09-10

**Authors:** Sidhartha Chaudhury, Wilawan Oransathit, Dutsadee Peerapongpaisarn, Wirote Oransathit, Chatchadaporn Thamnurak, Chantida Pradipol, Kirakarn Kirativanich, Sirigade Ruekit, Tanit Boonsiri, Yutthana Thanyathada, Anjali P. Sapre, Katelyn V. Bartlett, Melissa J. Martin, Paphavee Lertsethtakarn, Pattaraporn Vanachayangkul, Jeffrey R. Livezey, Daniel M. Boudreaux, Nattaya Ruamsap

**Affiliations:** ^1^Department of Bacterial and Parasitic Diseases, Walter Reed Army Institute of Research—Armed Forces Research Institute of Medical Sciences (WRAIR-AFRIMS), Bangkok, Thailand; ^2^Department of Microbiology, Phramongkutklao College of Medicine, Bangkok, Thailand; ^3^Clinical Microbiology Laboratory, Queen Sirikit Naval Hospital, Chonburi, Thailand; ^4^Multidrug-Resistant Organism Repository and Surveillance Network (MRSN), Walter Reed Army Institute of Research, Silver Spring, Maryland, United States

**Keywords:** ESKAPEE, antimicrobial resistance, wastewater, carbapenemase, ESBL, MDR, Thailand, one health

## Abstract

**Introduction:**

Wastewater surveillance has recently emerged as a promising method for AMR surveillance, but few studies have directly compared wastewater surveillance samples with clinical isolates and the clinical significance of wastewater surveillance for AMR bacteria is still unclear.

**Methods:**

We carried out monthly surveillance of hospital wastewater and surrounding community water at a tertiary hospital in Chonburi, Thailand from March to December 2024 and compared our findings with clinical isolates collected at the same hospital during the same period. For each wastewater sample, we isolated ESKAPEE pathogens, identified species by mass spectrometry, performed antimicrobial susceptibility testing (AST), followed by PCR testing of AMR genes and whole genome sequencing (WGS) on a subset of wastewater isolates and compared our results to clinical isolates.

**Results:**

We obtained 2,735 AMR isolates from untreated hospital wastewater, of which 1,550 were ESKAPEE pathogens including: *Klebsiella pneumoniae* (35.8%), *Enterobacter* spp. (397, 25.6%), *Escherichia coli* (24.9%), *Enterococcus faecium* (10.1%), *Acinetobacter baumannii* (2.7%), and *Pseudomonas aeruginosa* (0.8%). Based on AST data, we found that 85% *K. pneumoniae* isolates, 43% *A. baumannii* isolates, and 98% *E. coli* isolates, 62% *Enterobacter* spp. isolates, and 26% *E. faecium* isolates were classified as multi-drug resistant. We carried out hierarchical clustering of the AST data for a subset of 416 wastewater isolates along with 743 clinical isolates and found significant overlap in AST profiles of wastewater and clinical isolates. Using the clustering data, we selected a subset of 52 wastewater isolates with similar AST profiles to clinical isolates for WGS and identified 19 wastewater isolates that were highly genetically related (≤10 different alleles by cgMLST) to a clinical isolate, including 9 isolates with zero different alleles from closely related clinical isolates.

**Conclusion:**

Our results show that surveillance of untreated hospital wastewater is capable of identifying ESKAPEE that share similar drug resistance profiles, AMR genes, and clonal lineages found in the clinical isolates over the same time period. To our knowledge, this is one of the first studies to demonstrate a high level of genetic-relatedness between hospital wastewater and clinical isolates and demonstrate the clinical relevance of bacterial wastewater surveillance for MDR ESKAPEE pathogens.

## 1 Introduction

The increasing prevalence of antimicrobial resistance (AMR) bacteria presents a significant threat to global health. Recent estimates indicate that by 2050, AMR bacteria could result in over 10 million deaths world-wide (Collaborators, 2024). A recent study in 2019 estimated that 4.95 million deaths globally were attributed to AMR bacteria, of which ESKAPEE pathogens (*Enterococcus faecium, Staphylococcus aureus, Klebsiella pneumoniae, Acinetobacter baumannii, Pseudomonas aeruginosa, Enterobacter* species, and *Escherichia coli*) contributed to 3.57 million deaths (Antimicrobial Resistance, 2022). Deaths due to ESKAPEE pathogens are typically attributed to nosocomial infections acquired in healthcare setting, where they can present as multi-drug resistant (MDR) bacterial infections. Southeast Asia in particular has high rates of MDR bacterial infections. The Global Research on AntiMicrobial resistance (GRAM) project identified countries in Southeast Asia has having medium to high risk for MDR bacteria, particularly for *E. coli, A. baumannii*, and *K. pneumoniae*. Clinical AMR surveillance conducted in a tertiary hospital in Thailand in 2017-2018 identified high rates of MDR *A. baumannii, K. pneumoniae*, and *P. aeruginosa* with drug resistance to carbapenems, cephems, B-lactam inhibitors, fluoroquinolones, and aminoglycosides (Ruekit et al., 2022). Recent reports from Thailand have identified increasing prevalence of an extensively-drug resistant (XDR) *A. baumannii* strain resistant to all major drug classes except polymyxins, such as colistin (Khuntayaporn et al., 2021).

Wastewater surveillance has emerged as a powerful new tool to complement clinical surveillance of infectious diseases. This approach involves the collection of environmental water sources and testing those samples for the presence of pathogens of interest. While the clinical significance of wastewater surveillance for viral targets including respiratory viruses such as SARS-CoV2 (Peccia et al., 2020; D'Aoust et al., 2021; Li et al., 2022) and enteric viruses such as noroviruses and rotaviruses have been established (Aw and Gin, 2010; Wang et al., 2020) the clinical significance of wastewater surveillance for AMR bacterial pathogens is still not yet clear. Recent reviews noted that few wastewater surveillance studies for AMR bacteria involve the simultaneous collection of clinical samples needed to validate the clinical relevance of the wastewater data (Chau et al., 2022; Tiwari et al., 2022). Other challenges include the lack of standardization in wastewater analysis techniques, lack of knowledge on the durability and persistence of AMR bacteria in wastewater, and challenges in interpreting wastewater data (Chau et al., 2022; Tiwari et al., 2024).

In this study we conducted monthly wastewater surveillance from untreated hospital wastewater from a tertiary hospital in Chonburi, Thailand, where we were simultaneously conducting clinical surveillance for MDR bacteria with a focus on ESKAPEE pathogens. We used a culture-based approach in order to obtain wastewater isolates that we could directly compare with clinical isolates obtained at the same hospital. To assess the clinical relevance of the wastewater surveillance, we directly compared drug resistance profiles, AMR genes, and whole-genome sequencing data obtained from the wastewater isolates with the clinical isolates to determine the extent to which wastewater isolates captured phenotypic and genotypic characteristics of the clinical isolates.

## 2 Materials and methods

### 2.1 Ethic approval

This study was approved by the Research Ethic Committee, Naval Medical Department, Royal Thai Navy and Walter Reed Army Institute of Research (WRAIR), Silver Spring, MD, USA. WRAIR Human Subjects Protection Branch determined that this was non-human subjects research (NHSR) and all samples and data associated with clinical isolates was de-identified prior to transfer to AFRIMS.

### 2.2 Sample collection

We carried out surveillance of hospital wastewater effluent from Queen Sirikit Naval Hospital in Chonburi, Thailand for a ten month period from March to December 2024. An overview of the monthly testing process is shown in Supplementary Figure S1.

Water samples comprising of untreated hospital wastewater, treated hospital wastewater, and community water (local public water reservoir) were collected once a month (at the first week of each month) from March to December 2024, spanning both the dry and wet seasons. In each collection, water was manually sampled with volumes of 500 ml for untreated water and 5 L for treated water and community water as single-time point grab sample. These volumes were selected based on preliminary data on the bacterial burden in untreated wastewater, treated wastewater, and community water at these sites. Upon collection, the water samples were refrigerated during transportation to the laboratory to ensure sample integrity. Water samples were stored at 4 °C and processed within 24 h after collection.

### 2.3 Bacterial isolation

At the laboratory, water samples were filtered through 5 μM membrane (Merck Millipore, Burlington, MA) to remove the large particles. Afterwards, the filtrate was filtered through 0.45 μM membranes (Merck Millipore, Burlington, MA) and thoroughly rinsed the filtered membranes with 5 ml Phosphate Buffered Saline (PBS; Sigma-Aldrich, Burlington, MA). To isolate bacteria, the suspension was performed 3-fold dilution in PBS then 200 μl of the diluted suspension was spreaded onto the large petri-dish (diameter 150 mm) containing Brucella agar (Becton Dickinson, Franklin Lakes, NJ) with 5% sheep blood, CHROMagar^TM^ MRSA, mSuperCARBA, extended-spectrum beta-lactamase (ESBL), and VRE plates (CHROMagar™, Paris, France) and Eosin-methylene blue (EMB) agar plates (Becton Dickinson, Franklin Lakes, NJ) supplemented with 4 μg/ml colistin (Sigma-Aldrich, Burlington, MA). Brucella Blood agar plate, representing non-selective media, was used to identify total number of bacteria, while antibiotic plates were used to identify antibiotic-resistant bacteria. The plates were incubated under aerobic conditions at 37 °C for 24–48 h. Colony forming units (CFUs) were enumerated and converted into CFU/ml. In order to provide a relative measure for the degree of antimicrobial resistance in a given sample, a Resistance Index was calculated based on the ratio of the number of colonies found on a selective plate with the number of colonies found on a non-selective blood agar plate.


$$\text{Resistance Index=}\frac{\text{Bacteria on antibiotic agar plate }(\text{CFU/ml})\;}{\text{Bacteria on blood agar plate }(\text{CFU/ml})}$$

### 2.4 Mass spectrometry

Bacterial isolates on each antibiotic agar plates were further identified using MALDI-TOF (Bruker MALDI Biotyper system, Bremen, Germany) (Rahman et al., 2023). The lyophilized Bruker HCCA (4-Hydroxy-α-cyanocinnamic acid) matrix 2.5 mg was dissolved in 250 μl of standard solvent (50% acetonitrile, 47.5% water, and 2.5% trifluoroacetic acid). Mass spectrometry was performed and the bacterial isolates were analyzed and identified by Bruker MALDI Biotyper system and database (MALDI Biotyper 4.1.60 Software, Bremen, Germany). MALDI scoring method was used for interpretation. If the obtained score was ≥2.00, the genus and species identification were acceptable with a high confidence. If the score was 1.70–1.99, only the genus identification was acceptable. If the score was below the cut-off (1.70), the sample was recorded as no identification possible (Buchan et al., 2012).

### 2.5 Antimicrobial susceptibility testing

We carried out a two-step process for AST. As an initial screen, ESKAPEE isolates identified through mass spectrometry were subjected to AST by disk diffusion to identify isolates that show an MDR phenotype by testing against the following antibiotics: amikacin, gentamicin, trimethoprim/sulfamethoxazole, ciprofloxacin, cefepime, cefotaxime, ceftazidime, ceftriaxone, imipenem, meropenem, tetracycline, ampicillin, penicillin, and vancomycin. The subset of isolates that showed an MDR phenotype in the initial screen were then analyzed using the BD Phoenix^TM^ M50 system (Becton Dickinson, Franklin Lakes, NJ), using the NMIC/ID-504 panel for gram-negative ESKAPEE pathogens and PMIC/ID-95 panel for gram-positive ESKAPEE pathogens, according to the manufacturer's instructions (BD Diagnostics, Sparks, MD). Interpretations of resistance phenotypes from the disk diffusion method and the BD Phoenix^TM^ M50 system were made following CLSI guidelines (CLSI., 2024), using the guidelines for clinical isolates so that direct comparisons could be made between clinical and wastewater isolates.

### 2.6 PCR detection of AMR genes

Genomic DNA from bacterial isolates were extracted using the DNeasy Blood and Tissue Kit (QIAGEN, Hilden, Germany). The concentration and purity of the extracted DNA was using a Nanodrop 2000c Spectrophotometer (Thermo Scientific, Waltham, MA). The DNA, at a concentration between 1 and 10 ng/μl, was used as the template for PCR and real-time PCR and was stored at −20 °C until use. Real-time PCR assays were performed as described previously to determine the presence of carbapenemase antimicrobial resistance genes (*bla*_NDM_ and *bla*_KPC_) (Milillo et al., 2013) and methicillin resistance gene (*mecA*) (Mc Gann et al., 2013) using SensiFAST Probe No-ROX Mix (Bioline, London, UK) on CFX96 Touch Deep Well™ Real-Time PCR Detection System (Bio-Rad, Hercules, CA). Analysis was performed using Bio-Rad CFX Manager software version 3.1 (Bio-Rad, Hercules, CA). PCR assays were performed as described previously to determine the presence of ESBL genes (Dallenne et al., 2010), carbapenemase genes (*bla*_OXA − 23_, *bla*_OXA − 24_, *bla*_OXA − 51_, *bla*_OXA − 58_) (Feldgarden et al., 2019), colistin resistance genes (*mcr-1* to *mcr-9*) (Rebelo et al., 2018; Borowiak et al., 2020), and vancomycin resistance genes (*vanA, vanB, vanC, vanD, vanE*, and *vanG*) (Pourcel et al., 2017) using the AmpliTaq^®^ Gold DNA polymerase (Thermo Fisher Scientific, Waltham, MA) on Mastercycler^®^ nexus (Eppendorf, Hamburg, Germany). Primers for *mcr-10* were designed based on full-length *mcr-10* gene (GenBank accession number MT468575) using Primer3Plus (https://www.primer3plus.com/index.html). The sequences are as follows: MCR-10_133F (5′-GCAATAACCCGACGCTGAAC-3′) and MCR-10_133R (5′-GTAACGCGCCTTGCATCATC-3′). These primers were incorporated into *mcr-6* to *mcr-9* multiplex (Borowiak et al., 2020).

### 2.7 Clinical isolates

We obtained 743 clinical isolates from the Microbiology Laboratory at Queen Sirikit Naval Hospital (QSH) as part of on-going AMR surveillance effort between AFRIMS and QSH between March and December of 2024. Bacteria isolated from clinical samples (e.g., pus, urine, rectal swabs, sputum, and blood) that were collected as part of routine medical care in both inpatient and outpatient settings were tested at QSH using the VITEK2 system (bioMérieux, Durham, NC). Isolates that were determined to be ESKAPEE organisms and classified as MDR based on non-susceptibility to at least one drug in three or more antimicrobial drug classes (Magiorakos et al., 2012) were selected and transferred to AFRIMS for confirmation of both speciation and AST phenotypes by the BD Phoenix^TM^ M50 system. The pure isolates were grown on tryptic soy agar plates and Brucella Blood agar plates in preparation for analyses.

### 2.8 Whole genome sequencing and bioinformatics analysis

Wastewater isolates (*n* = 52) were selected for whole genome sequencing (WGS) based on whether they had a similar AST phenotype profile as clinical isolates collected from the same hospital. This was done to maximize the use of limited WGS resource toward identifying wastewater isolates of high clinical relevance. As part of on-going clinical surveillance at QSH, 127 clinical MDR ESKAPEE isolates were also selected for WGS. DNA was extracted using the DNeasy UltraClean Microbial Kit (QIAGEN, Hilden, Germany). Library construction was performed with KAPA Library Quantification Kit (Roche Dianostics Corporation, Indianapolis, IN) for sequencing on an Illumina MiSeq Benchtop sequencer (Illumina, San Diego, CA) as previously described (Galac et al., 2020). Minimum thresholds for contig size and coverage were set at 200 bp and 49.5+, respectively. Assembled sequences were annotated using Prokka v1.14.6 (Seemann, 2014).

Species identification was determined using Kraken2 (v2.0.8-β) and *in silico* ST detection was identified for all isolates using species specific schemes for *E. coli, Enterobacter* spp., *A. baumannii, K. pneumoniae, P. aeruginosa*, and *S. aureus* (www.pubmlst.org). Antimicrobial resistance genetic determinants were annotated using AMRFinderPlus (Feldgarden et al., 2019) and ARIBA (Hunt et al., 2017). Minimum spanning trees (MST) were generated using core genome MLST (cgMLST) with species specific cgMLST schemes (https://www.cgmlst.org/ncs). Isolates with 0-10 allelic differences are considered highly genetically related based on prior studies on epidemiological outbreak detection using cgMLST (Coles, 1988; Leeper et al., 2023). This genome-based genetic relatedness analysis was performed on the 52 wastewater isolates and 127 clinical isolates collected during the study period in addition to >1,600 clinical isolates collected from the same hospital and sequenced since September 2022.

### 2.9 Data analysis

Descriptive statistics was used to determine significant differences between bacterial burden from different water sources. Phenotypic analysis of AST profiles was analyzed using the phenotype classification as determined by the BD Phoenix^TM^ M50 system. We applied a clustering approach for identifying groups of isolates with similar AST phenotypes that uses a pairwise distances between AST phenotype profiles of each isolate identify groups of isolates with similar AST profiles based on a method developed by Sanderson et al. (2019). First each isolate, its AST phenotype profile was represented as a character string with the number of digits and position of each character corresponding to a drug in the AST panel, with the value of the character being “1” if the phenotype for that isolate for that drug was R, and “0” if the phenotype was S or I. AST profiles of gram-negative bacteria were represented by an 18-digit string while profiles for gram-positive bacteria were represented by a 19-digit string, corresponding to the number of antibiotics in the BD Phoenix^TM^ M50 testing panels. A distance between the AST phenotype profiles of two isolates was calculated as the Hamming Distance (Hamming, 1950) between their AST phenotype character strings, defined as the sum of mismatches between the two strings. For each organism, a pairwise distance matrix for all isolates was generated and used for hierarchical clustering using the *hclust* algorithm in the R statistical software platform. AST phenotype clusters were defined using the *cutree* function with the height parameter set to 1. Supplementary Figure S2 illustrates numbers of ESKAPEE in untreated wastewater characterized by MALDI-TOF through whole genome sequencing analysis.

## 3 Results

### 3.1 AMR bacterial burden in wastewater

**Figure 1:**
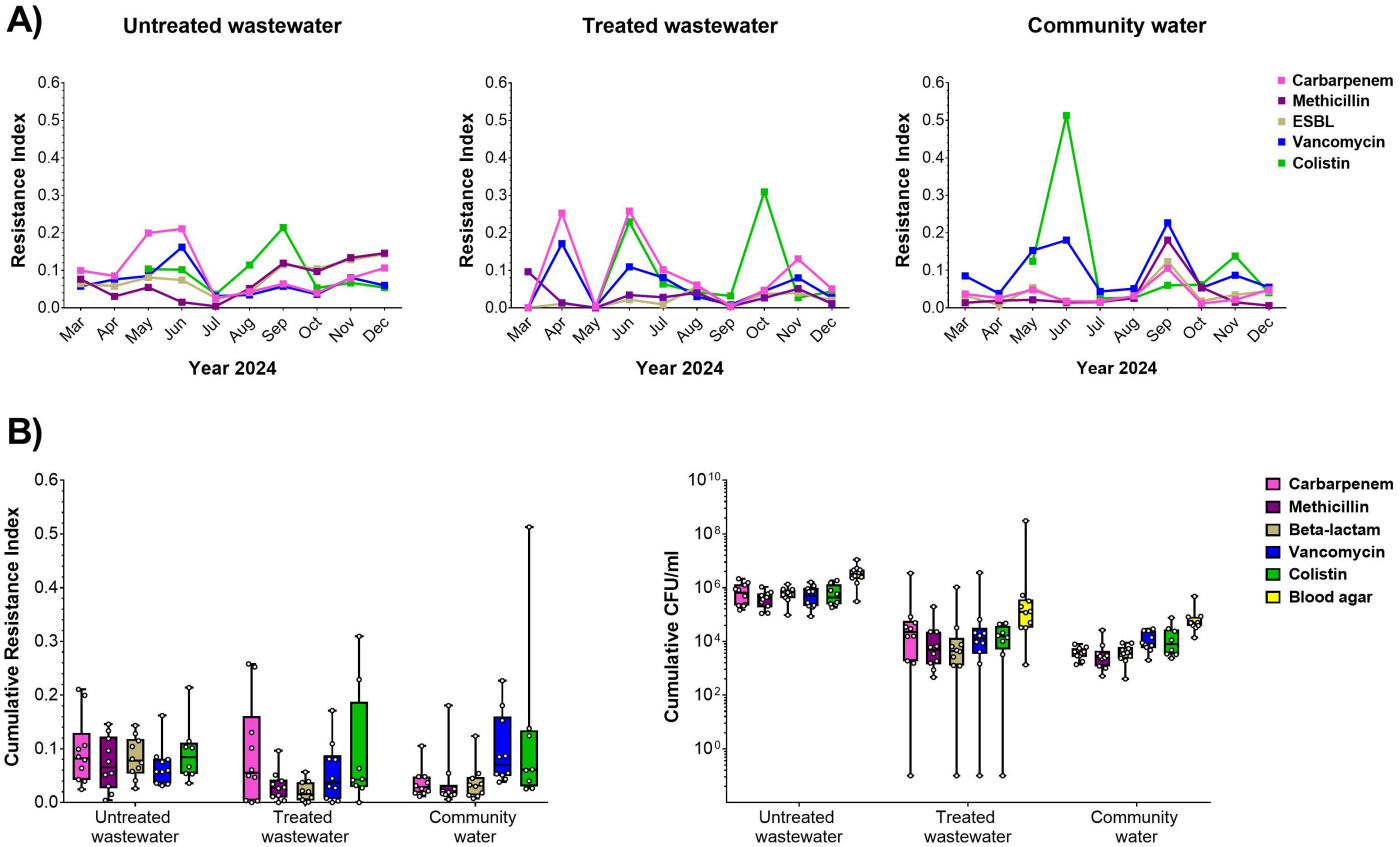
Antibiotic resistance in hospital water sources. (A) Resistance index against carbapenem (pink), methicillin (magenta), ESBL (gray), vancomycin (blue), and colistin (green) relative to overall biomass in the non-selective plate for untreated (left), treated (center), and community water (right) sources for monthly sample collections during the study period. (B) Cumulative resistance index shown across the entire study period (left) and biomass (CFU/ml, right) for untreated wastewater, treated wastewater, and community water.

Water samples were collected monthly from the hospital, filtered, cultured on selective and non-selective media and assessed by Resistance Index as a relative measure of drug resistance in the bacterial biomass (Figure 1A). We found that the Resistance Index varied significantly from month to month. For carbapenems, we found, in general, that untreated and treated water samples tended to have higher resistance than community water. For vancomycin, interestingly, we found that community water tended to have higher resistance. For ESBL resistance, we did find that untreated water had significantly higher resistance than treated water. For colistin, the peaked resistance was found in particular month such as September, October, and June for untreated water, treated water, and community water, respectively. Overall, we found that, in general, untreated water had higher cumulative CFU/ml than treated water and community water for all the selective plates (Figure 1B, Supplementary Table S1), showing that untreated hospital water was enriched for antibiotic resistance bacteria.

### 3.2 ESKAPEE isolates and drug resistance

**Figure 2:**
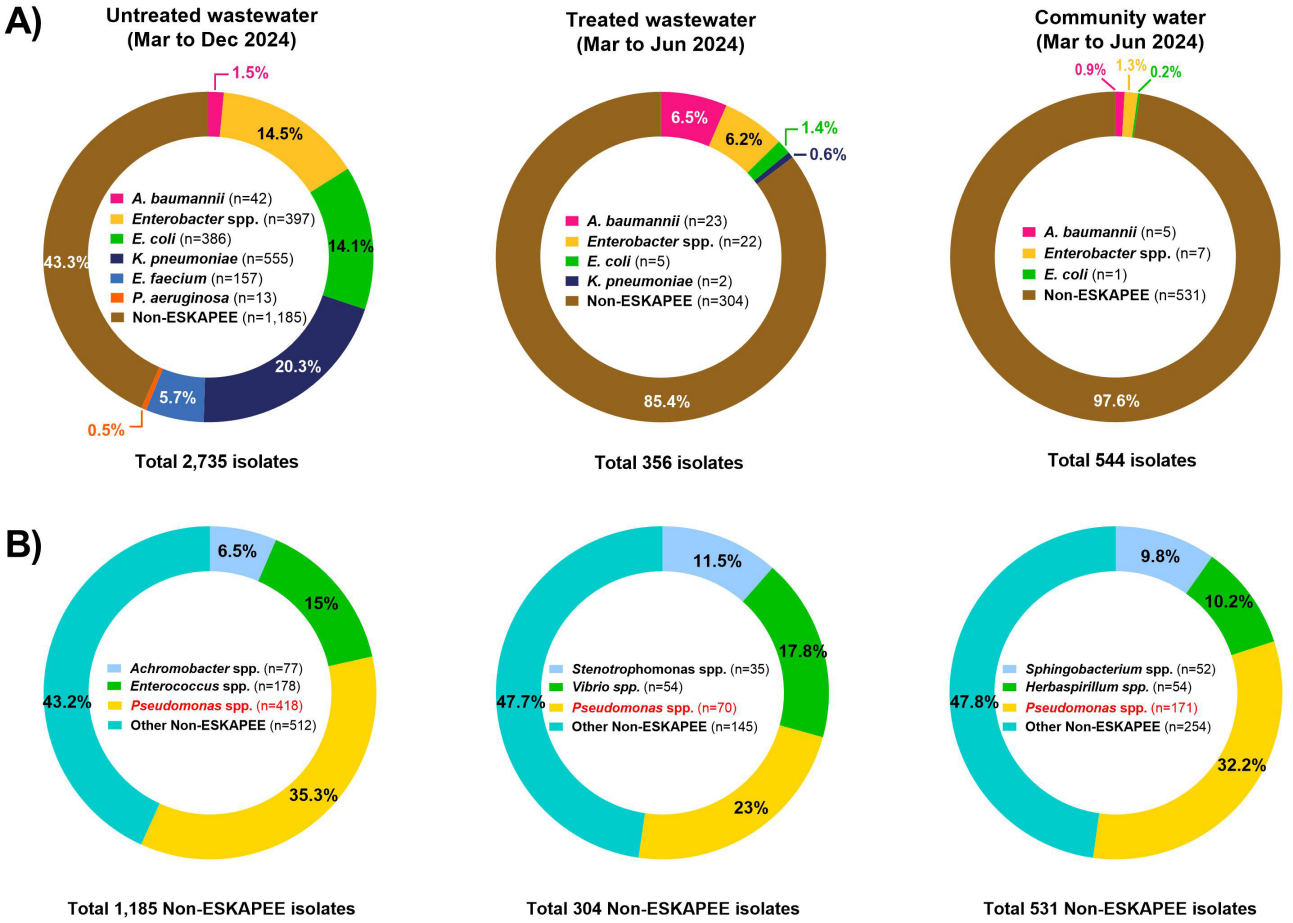
Profile of ESKAPEE and non-ESKAPEE pathogens in wastewater. **(A)** MALDI-TOF was used to carry out speciation of drug resistant isolates collected from untreated hospital wastewater (left), treated hospital wastewater (center) and community water sources. ESKAPEE pathogens (colored) and non-ESKAPEE organisms (brown) are shown. **(B)** Species profile of non-ESKAPEE pathogen is shown below for all three water sources.

We selected drug resistant isolates from selective plates for speciation using MALDI-TOF mass spectrometry. Figure 2 shows the profile of both ESKAPEE and non-ESKAPEE pathogens in untreated wastewater, treated wastewater, and community water from the hospital. Overall, we found that a majority of drug resistant isolates from untreated hospital wastewater (1,550 of 2,735, 56.7%) were ESKAPEE pathogens, with *K. pneumoniae, E. coli*, and *Enterobacter* spp. being the most common, accounting for 48.9% of drug resistant isolates. By contrast, treated wastewater had significantly fewer ESKAPEE isolates (14.6%, 52 of 356 isolates), predominantly *A. baumannii*, and *Enterobacter* spp. Likewise, we found that ESKAPEE isolates represented a very small fraction (13 of 544 isolates, 2.4%) of drug resistant isolates in community water. Non-ESKAPEE isolates varied based water source with *Achromobacter* isolates being the most common in untreated hospital wastewater, S*tenotrophomone* isolates being the most common in treated hospital wastewater and *Sphingobacter* isolates being the most common in community water, with *Pseudomonas* spp. (non-*aeruginosa*) being the second most common in all three sources (Figure 2).

**Figure 3:**
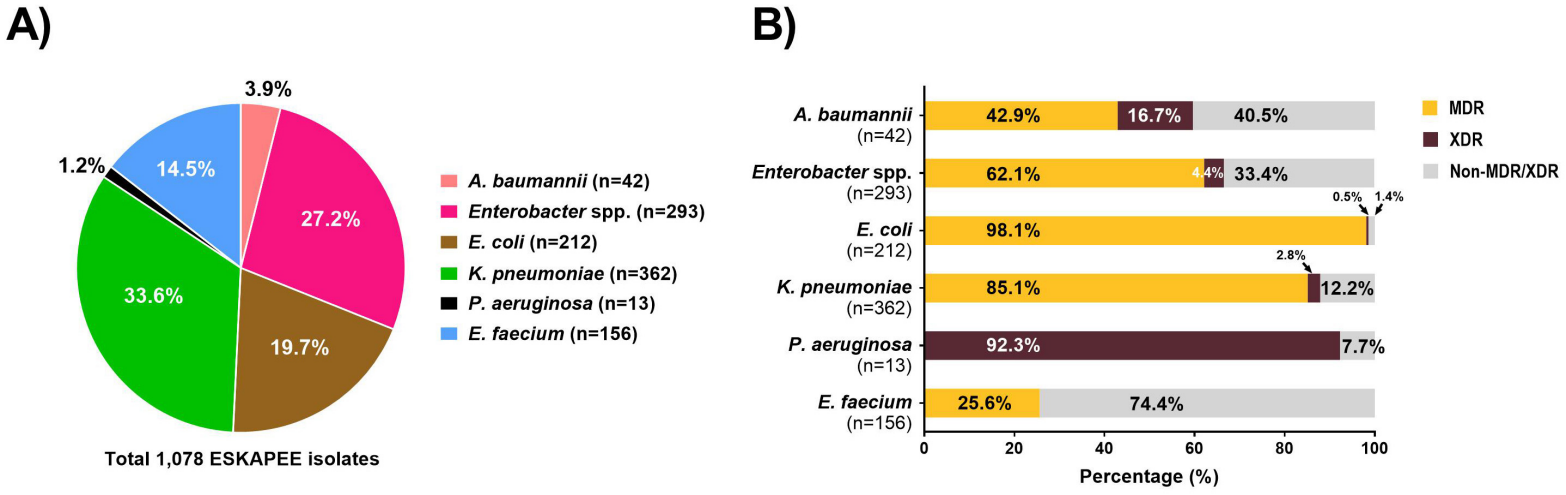
Antimicrobial susceptibility testing of ESKAPEE isolates from untreated hospital wastewater. **(A)** Profile of ESKAPEE isolates that were selected for AST using the disk diffusion methods. **(B)** Classification of ESKAPEE pathogens as MDR (orange) and XDR (maroon) based on the AST data.

For each ESKAPEE isolate from the untreated hospital wastewater, we carried out AST using disk diffusion followed by the automated BD Phoenix^TM^ M50 system. Overall, 1,550 isolates were identified as ESKAPEE organisms by MALDI-TOF. Of that, 1,078 isolates were selected for AST by disk diffusion, and 367 of the isolates exhibiting MDR pattern were characterized by the BD Phoenix^TM^ M50 system (Figure 3). The most common drug resistant ESKAPEE isolates based on disk diffusion were *Enterobacter* spp., *E. coli*, and *K. pneumoniae*. Based on the BD Phoenix^TM^ M50 data, we classified isolates as MDR (non-susceptible to at least three classes of antibiotics), and XDR (non-susceptible to all but one or fewer classes of antibiotics). We found that most of the ESKAPEE isolates we collected (799 of 1,078, 74%) were MDR, with almost all (12 of 13, 92.3%) the *P. aeruginosa* isolates being XDR.

The breakdown of ESKAPEE isolates from hospital wastewater based on resistance types is shown in Supplementary Figure S3. Overall, we found that high rates of carbapenemase producers among *A. baumannii, Enterobacter* spp., *K. pneumoniae*, and *P. aeruginosa* isolates (60-90%). We found high rates of ESBL producers among *E. coli* and *K. pneumoniae* isolates (~70%). For colistin resistance, we observed relatively low resistance in *E. coli* and *K. pneumoniae* isolates (20–30%) but high reistance among *Enterobacter* spp. isolates (55%). Finally, we found that 24% of *E. faecium* isolates showed vancomycin resistance.

Our approach was able to capture gram-positive ESKAPEE pathogens, specifically 35 isolates of *E. faecium*. However, among MDR clinical isolates collected during the same period, the only gram-positive pathogen that was identified was *S. aureus* (17 out of 743 clinical isolates). Because of the low prevalance of gram-positive MDR pathogens among clinical isolates and the lack of overlap between gram-positive ESKAPEE pathogens in the clinical and wastewater samples (*E. faecium* in wastewater vs. *S. aureus* in clinical samples), the subsequent analyses focus on the following gram-negative ESKAPEE pathogens: *A. baumannii, E. cloacae, K. pneumoniae, P. aeruginosa* and *E. coli*.

### 3.3 AST phenotype profiles

**Figure 4:**
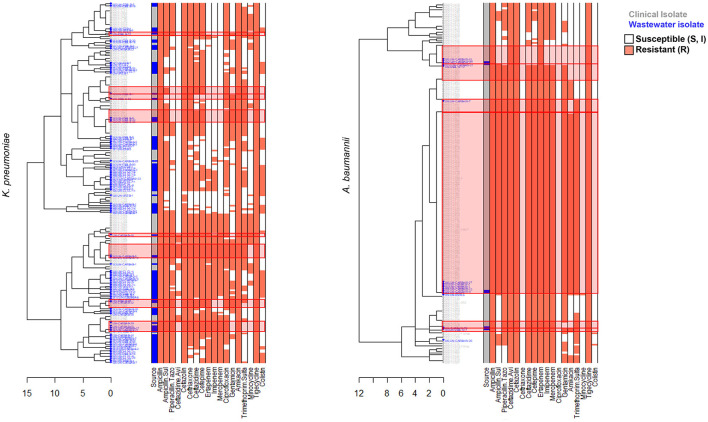
AST phenotype profiles for *K. pneumoniae* and *A. baumannii*. Dendrograms shown following hierarchical clustering of the AST phenotype profiles from wastewater (blue) and clinical (gray) isolates for two ESKAPEE pathogens. AST phenotypes are shown for resistant (orange) and sensitive/intermediate (white) phenotypes for the antibiotic panel. AST phenotype clusters that include both clinical and wastewater isolates are highlighted in red rectangles.

**Figure 5:**
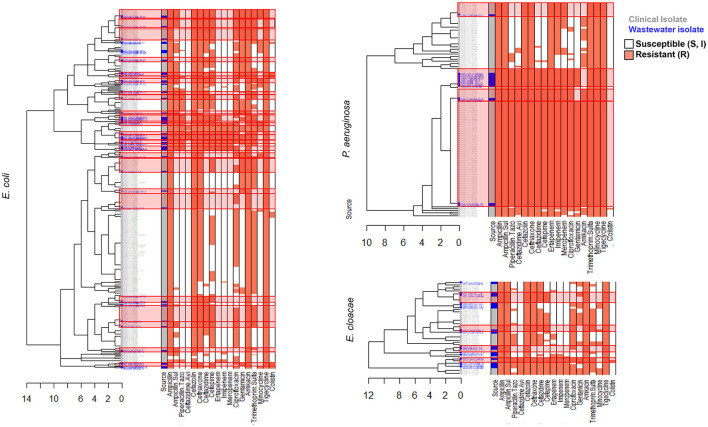
AST phenotype profiles for E. coli, P. aeruginosa, and E. cloacae. Dendrograms shown following hierarchical clustering of the AST phenotype profiles from wastewater (blue) and clinical (gray) isolates for two ESKAPEE pathogens. AST phenotypes are shown for resistant (orange) and sensitive/intermediate (white) phenotypes for the antibiotic panel. AST phenotype clusters that include both clinical and wastewater isolates are highlighted in red rectangles.

We carried out AST using disk diffusion method followed by automated identification and testing for a subset of 416 wastewater isolates, of which 251 were confirmed as ESKAPEE pathogens. We combined this data with BD Phoenix^TM^ M50 data from 743 clinical ESKAPEE isolates and generated AST profiles for each isolate based on whether they were sensitive (S or I) or resistant (R) to each antibiotic in the drug panel (Sanderson et al., 2019). We then carried out hierarchical clustering to define clusters of isolates with similar AST profiles (Figures 4, 5). Overall, we found that for five ESKAPEE pathogens: *K. pneumoniae, A. baumannii, E. coli, P. aeruginosa*, and *E. cloacae*, we found many clusters where both clinical and wastewater isolates had similar or identical AST profiles.

We sought to determine the degree to which the wastewater isolates captured the diversity of clinical isolates (Table 1) by determining what percentage of clinical isolate clusters (representing unique clinical phenotypes) contained a wastewater isolate with a matching AST phenotype. For *E. coli*, we found that almost 50% of clinical AST phenotype clusters included a wastewater isolate with the same AST phenotype. For *A. baumannii, K. pneumoniae, E. cloacae* that number was 35, 28, and 27%, respectively, compared to 17% for *P. aeruginosa*. Overall, this shows that while some clinical AST phenotype patterns are captured in the AST phenotype profiles of wastewater isolates, many clinical phenotypes are still missing.

**Table 1:** AST phenotype diversity.

**Pathogens**	**# of wastewater-only clusters**	**# of clinical-only clusters**	**# of shared clusters**	**% of clinical clusters with wastewater isolates**
*E. coli*	12	32	25	44%
*K. pneumoniae*	47	23	9	28%
*A. baumannii*	1	19	6	35%
*P. aeruginosa*	0	14	3	18%
*E. cloacae*	7	11	4	27%

We also sought to determine the degree of coverage that wastewater isolates provide in capturing high-frequency clinical phenotypes (Table 2), by assessing what proportion of all clinical isolates have a wastewater isolate with the same AST phenotype. We found that for *A. baumannii* and *P. aeruginosa*, the coverage was relatively high, with 72 and 73% of clinical isolates being represented by a phenotypically matching wastewater isolate, respectively. For *E. coli* and *K. pneumoniae*, there was moderate coverage with 38 and 32% coverage of clinical isolates, while for *E. cloacae* there was very low coverage of 10%. When we assessed what proportion of wastewater isolates have a clinical isolate of the same AST phenotype, we found that for *A. baumannii* and *P. aeruginosa*, coverage is very high at 94 and 100%, respectively, suggesting that most or all of wastewater isolates for these two organisms may be clinically relevant. For *E. coli* there was more moderate coverage of 70%, while it was lower for *E. cloacae* and *K. pneumoniae*, at 38 and 17% coverage, respectively.

**Table 2:** Clinical isolate coverage.

**Pathogen**	**Isolates in wastewater-only clusters**	**Isolates in clinical-only clusters**	**Clinical isolates in shared clusters**	**Wastewater isolates in shared clusters**	**% of clinical isolates in Shared cluster (95% CI)**	**% of wastewater isolates in shared clusters (95% CI)**
*E. coli*	19	175	109	42	38% (33, 44)	70% (56, 80)
*K. pneumoniae*	86	69	33	17	32% (23, 42)	17% (10, 25)
*A. baumannii*	1	49	131	16	73% (67, 79)	94% (71, 100)
*P. aeruginosa*	0	30	77	13	72% (62, 80)	100% (75, 100)
*E. cloacae*	10	22	7	6	24% (10, 44)	38% (15, 65)

CI, confidence interval.

### 3.4 AMR gene profile

We carried out PCR-based testing of AMR genes in the ESKAPEE isolates from wastewater used for AST testing and compared it to AMR genes identified in clinical samples to determine the degree to which AMR genes identified in wastewater isolates recapitulated AMR genes identified in clinical isolates. A high degree of overlap would indicate that wastewater surveillance can be used to monitor the emergence of clinically relevant AMR genes in ESKAPEE pathogens. Table 3 shows the list of AMR genes tested in gram-negative ESKAPEE pathogens which shows which AMR genes were identified in at least one wastewater isolate (denoted by a “+”), and then the proportion of clinical isolates that also have that same gene. For *K. pneumoniae*, we found 80% sensitivity and 100% specificity in wastewater AMR genes identifying from clinical isolates, notably for *bla*_NDM_, *bla*_TEM_, *bla*_SHV_, *bla*_OXA_, and *bla*_CTX − M−1_ genes which showed high prevalence in clinical samples.

**Table 3:** AMR gene detection and prevalence.

**AMR genes**	***K. pneumoniae***		***P. aeruginosa***		***A. baumannii***		***E. coli***		***E. cloacae***	
	**Wastewater (*****n*** = **87)**	**Clinical (*****n*** = **105)**	**Wastewater (*****n*** = **4)**	**Clinical (*****n*** = **107)**	**Wastewater (*****n*** = **12)**	**Clinical (*****n*** = **180)**	**Wastewater (*****n*** = **39)**	**Clinical (*****n*** = **284)**	**Wastewater (*****n*** = **19)**	**Clinical (*****n*** = **29)**
*bla*_KPC_	-	0.01	-	0.00	**-**	0.00	+	0.00	-	0.00
*bla*_NDM_	+	0.14	+	0.61	**+**	0.42	+	0.05	+	0.07
*mcr-1* to *mcr-9*	+	0.01	-	0.00	**-**	0.00	+	0.03	-	0.00
*mcr-10*	-	0.00	-	0.00	**-**	0.00	-	0.00	+	0.00
*bla*_TEM_	+	0.61	-	0.00	**+**	0.41	+	0.39	+	0.41
*bla*_SHV_	+	0.74	-	0.00	**-**	0.00	-	0.01	-	0.14
*bla*_OXA_	+	0.28	-	0.01	**-**	0.00	+	0.16	-	0.28
*bla*_OXA − 48_	-	0.19	-	0.00	**-**	0.00	+	0.01	-	0.00
*bla*_CTX − M−1_	+	0.69	-	0.00	**-**	0.00	+	0.40	+	0.48
*bla*_CTX − M−2_	-	0.00	-	0.00	**-**	0.00	-	0.00	-	0.00
*bla*_CTX − M−9_	+	0.07	-	0.00	**-**	0.00	+	0.44	-	0.00
*bla*_CTX − M−8/25_	+	0.05	-	0.00	**-**	0.00	+	0.00	+	0.00
*bla*_ACC_	-	0.00	-	0.00	**-**	0.00	-	0.00	-	0.00
*bla*_FOX_	-	0.00	-	0.00	**-**	0.00	-	0.00	-	0.00
*bla*_MOX_	-	0.00	-	0.00	**-**	0.00	-	0.00	-	0.00
*bla*_DHA_	+	0.10	-	0.02	**-**	0.00	-	0.07	-	0.00
*bla*_CMY_ (*bla*_CIT_)	-	0.00	-	0.00	**-**	0.00	+	0.05	-	0.03
*bla*_EBC_	-	0.00	-	0.00	**-**	0.00	-	0.00	-	0.07
*bla*_GES_	**+**	0.01	+	0.01	**-**	0.00	+	0.00	+	0.00
*bla*_PER_	-	0.00	-	0.00	**-**	0.02	-	0.00	-	0.03
*bla*_VEB_	+	0.03	+	0.18	**-**	0.01	+	0.00	+	0.07
*bla*_IMP_	-	0.00	-	0.45	**-**	0.00	-	0.00	-	0.00
*bla*_VIM_	-	0.00	-	0.00	**-**	0.00	-	0.00	-	0.00
*bla*_KPC_	-	0.00	-	0.00	**-**	0.00	+	0.00	-	0.00
*bla*_OXA − 23_	-	0.00	-	0.00	**+**	0.87	-	0.00	-	0.00
*bla*_OXA − 24_	-	0.00	-	0.00	**-**	0.01	-	0.00	-	0.00
*bla*_OXA − 51_	-	0.00	-	0.00	**+**	0.92	-	0.00	-	0.00
*bla*_OXA − 58_	-	0.00	-	0.00	**-**	0.04	-	0.00	-	0.00
*bla*_OXA − 143_	-	0.00	-	0.00	**-**	0.00	-	0.00	-	0.00
*bla*_OXA − 69_	-	0.00	-	0.00	**+**	0.93	-	0.00	-	0.00
Sensitivity†	85%		60%		56%		80%		33%	
Specificity†	100%		100%		100%		62%		57%	

†, sensitivity and specificity are calculated based on ability for detection of presence of an AMR gene in wastewater sample.

(+), to predict non-zero prevalence (>0.0) of that same AMR gene in clinical isolates for each ESKAPEE pathogen.

For *P. aeruginosa* and *A. baumannii*, we found that analysis of wastewater isolates had moderate sensitivity (~60%) and high specificity (100%) in identifying AMR genes from their respective clinical isolates, notably for high-prevalence genes *bla*_NDM_ and *bla*_VEB_ in *P. aeruginosa* and *bla*_NDM_, *bla*_TEM_, *bla*_OXA − 23_, *bla*_OXA − 51_, and *bla*_OXA − 69_ in *A. baumannii*. For *E. coli*, we found that wastewater isolates had high sensitivity (80%) but relatively low specificity (62%), with a number of AMR genes identified in wastewater isolates that were absent in clinical isolates, including *bla*_KPC_, *bla*_CTX − M−8/25_, *bla*_GES_, *bla*_VEB_, and *bla*_KPC_. For these four ESKAPEE pathogens, it is notable that there were few cases of a highly prevalent AMR gene in a clinical isolate that was not found in the wastewater isolate—the *bla*_IMP_ gene in clinical *P. aeruginosa* isolates (45% prevalence in clinical samples) and *bla*_OXA − 48_ family genes in *K. pneumoniae* (19% prevalence in clinical samples).

For *E. cloacae*, we found low sensitivity (33%) and moderate specificity (57%), with numerous clinical AMR genes missing in the wastewater samples including *bla*_SHV_, *bla*_OXA_, *bla*_CMY_, *bla*_EBC_, and *bla*_PER_. It is important to note that in most of the cases were an AMR gene from clinical isolate was absent in wastewater isolates, it had relatively low prevalence in the clinical samples, suggesting that the wastewater surveillance may have been limited by low sample size.

### 3.5 Whole-genome sequencing

**Figure 6:**
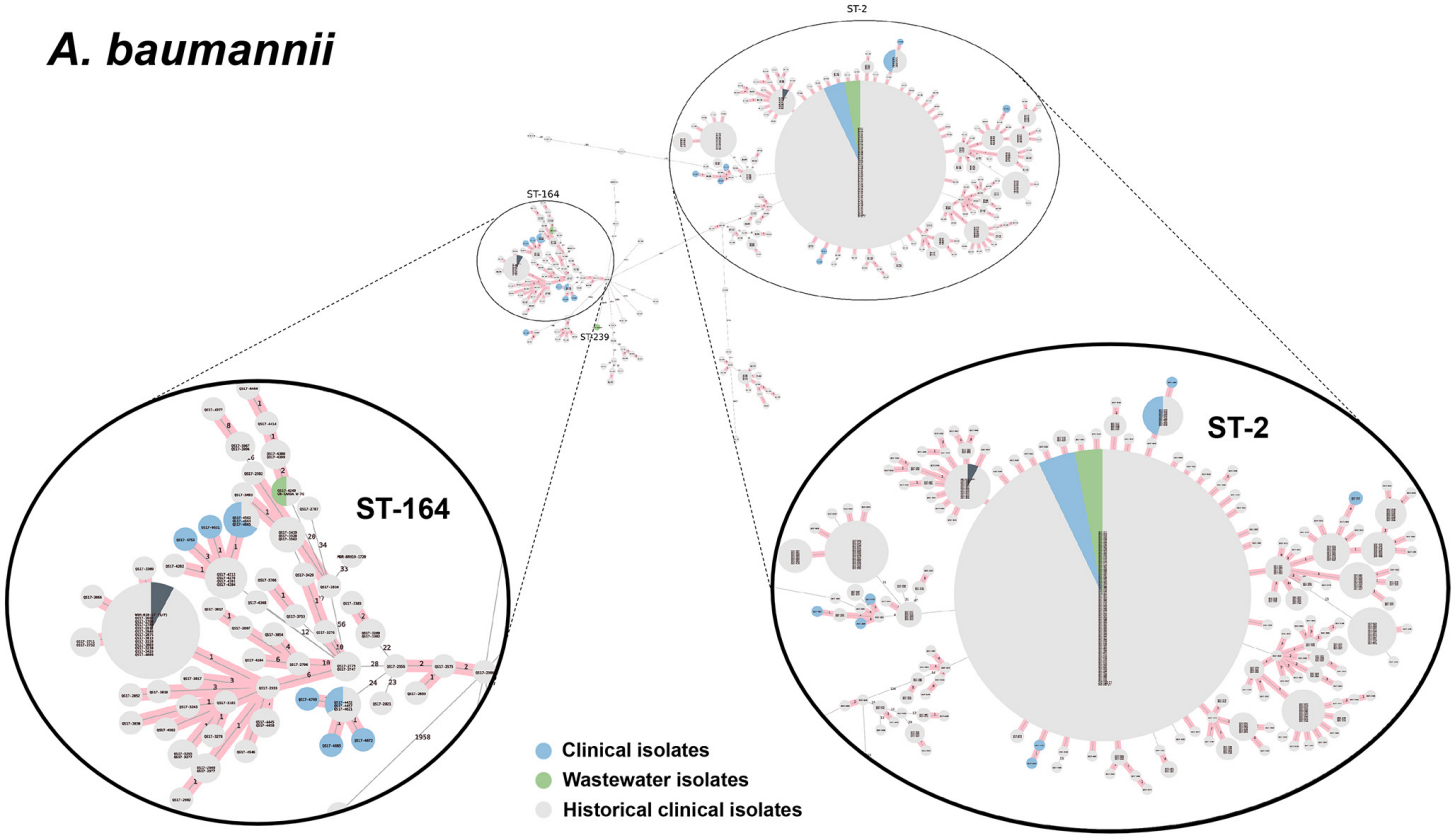
cgMLST-based minimum spanning trees of *A. baumannii* identified highly genetically related clinical and wastewater isolates. The size of each node corresponds to the number of isolates in that node with 0 allelic differences. The number in the line connecting two isolates indicates the number of allelic differences. Red shading indicates clusters of highly genetically related isolates (≤10 allelic differences). We also included historical data from clinical isolates collected prior to this study (gray). Clonal lineages representing clinical/wastewater clusters are maximized, circled and ST is labeled.

**Figure 7:**
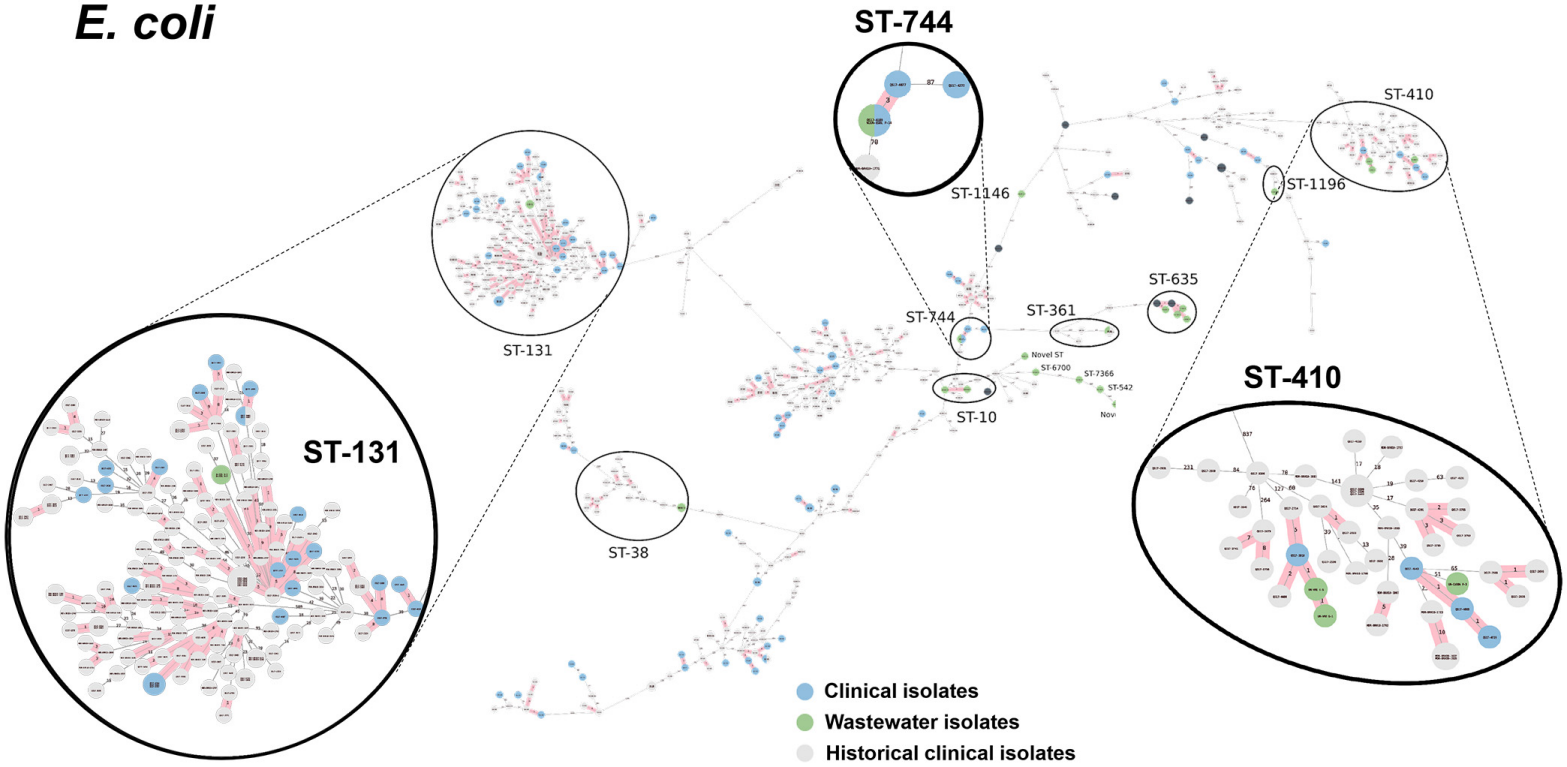
cgMLST-based minimum spanning trees of *E. coli* identified highly genetically related clinical and wastewater isolates. The size of each node corresponds to the number of isolates in that node with 0 allelic differences. The number in the line connecting two isolates indicates the number of allelic differences. Red shading indicates clusters of highly genetically related isolates (≤10 allelic differences). We also included historical data from clinical isolates collected prior to this study (gray). Clonal lineages representing clinical/wastewater clusters are maximized, circled and ST is labeled.

**Figure 8:**
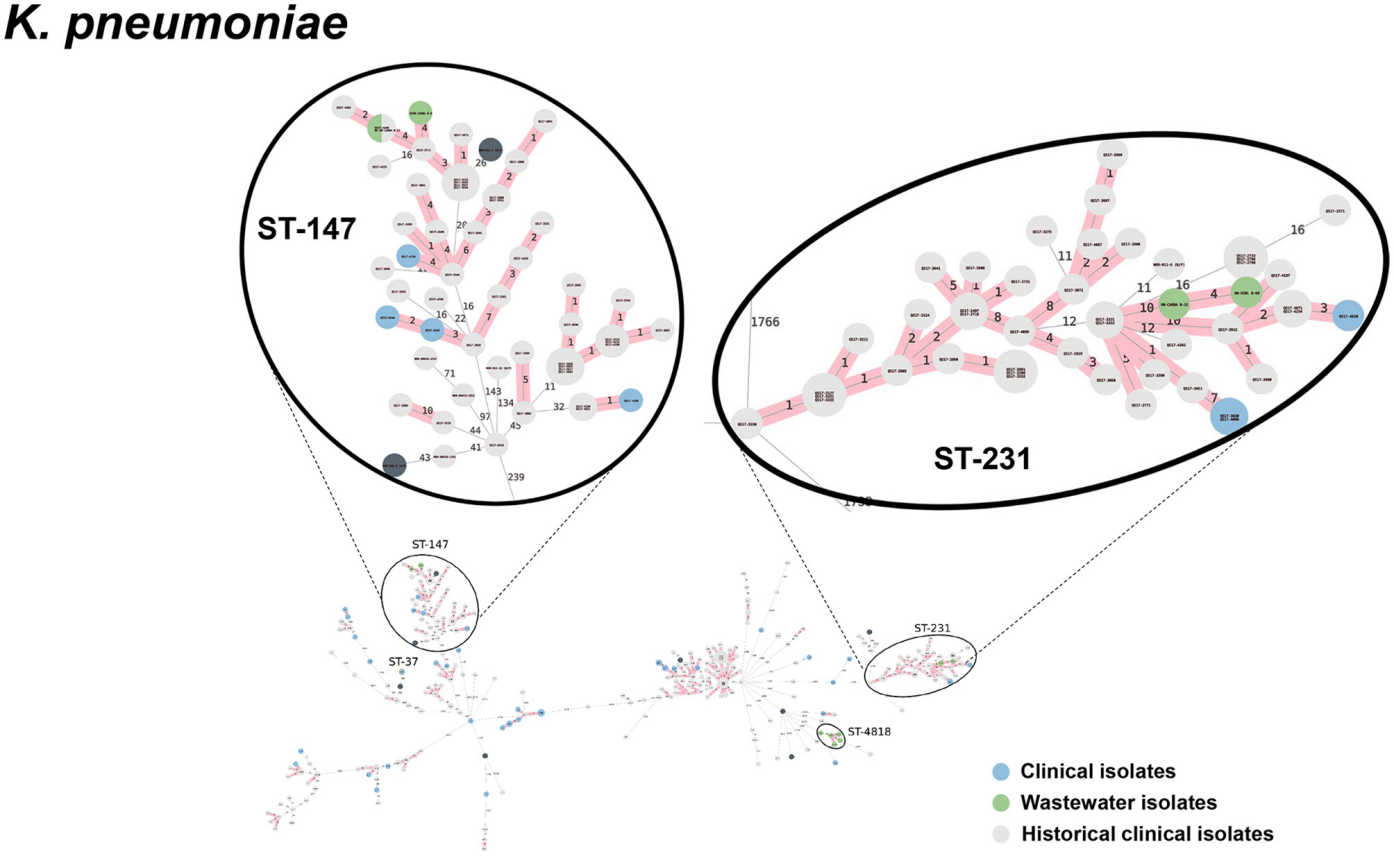
cgMLST-based minimum spanning trees of *K. pneumoniae* identified highly genetically related clinical and wastewater isolates. The size of each node corresponds to the number of isolates in that node with 0 allelic differences. The number in the line connecting two isolates indicates the number of allelic differences. Red shading indicates clusters of highly genetically related isolates (≤10 allelic differences). We also included historical data from clinical isolates collected prior to this study (gray). Clonal lineages representing clinical/wastewater clusters are maximized, circled and ST is labeled.

**Figure 9:**
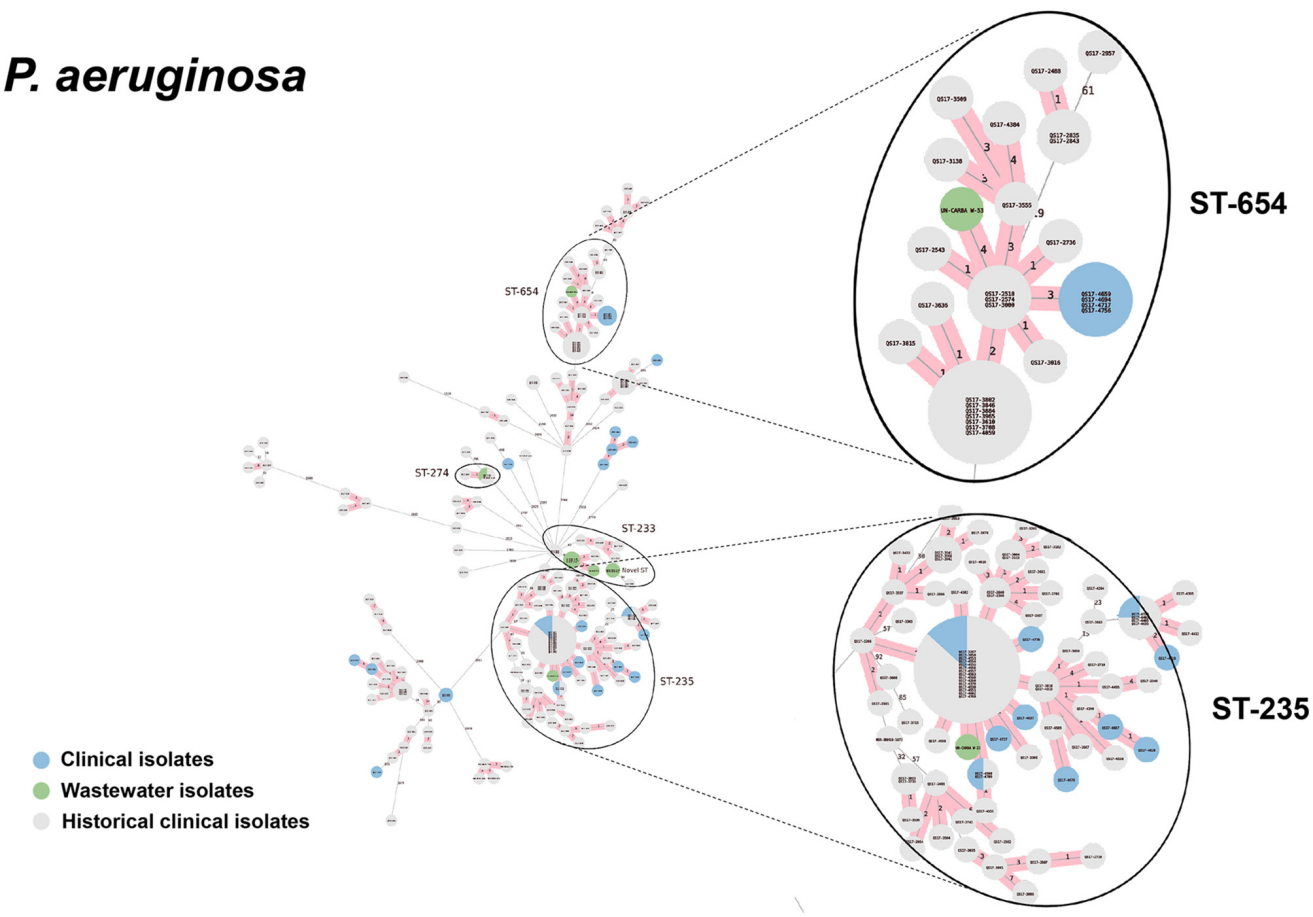
cgMLST-based minimum spanning trees of *P. aeruginosa* identified highly genetically related clinical and wastewater isolates. The size of each node corresponds to the number of isolates in that node with 0 allelic differences. The number in the line connecting two isolates indicates the number of allelic differences. Red shading indicates clusters of highly genetically related isolates (≤10 allelic differences). We also included historical data from clinical isolates collected prior to this study (gray). Clonal lineages representing clinical/wastewater clusters are maximized, circled and ST is labeled.

We selected a subset of 52 ESKAPEE isolates for WGS by selecting wastewater isolates that were found in the same AST phenotype cluster as clinical isolates including *E. coli* (*n* = 22), *K. pneumoniae* (*n* = 10), *P. aeruginosa* (*n* = 9), *A. baumannii* (*n* = 5), *E. faecium* (*n* = 3), and *E. cloacae* (*n* = 3). Using cgMLST analysis, we found that for *A. baumannii* (Figure 6), *E. coli* (Figure 7), *K. pneumoniae* (Figure 8), and *P. aeruginosa* (Figure 9), wastewater isolates have the same sequence type as clinical isolates, and were highly genetically related. For *E. cloacae*, the sequenced wastewater isolates were not genetically related to clinical isolates (Supplementary Figure S4) suggesting that any similarities in AST profiles in this pathogen are not reflective of relatedness.

Table 4 shows a summary of sequenced wastewater isolates that were found to be of the same sequence type (ST) as either a clinical isolate collected during the study period, or a historical clinical isolate collected earlier at the same site. For *E. coli*, we found 7 of 22 sequenced wastewater isolates were in the same ST as a clinical isolate, including an ST744 isolate and ST361 isolate where there were no allelic differences found between the wastewater and clinical isolates, indicating extremely high genetic relatedness. AST phenotypes for these shared strains include MDR and XDR phenotypes. For *K. pneumoniae*, we found 5 of 10 sequenced wastewater isolates were in the same ST as a clinical isolate, including two ST147 isolates and one ST37 isolate which also showed no allelic differences to a clinical isolate.

**Table 4:** Genetically related wastewater and clinical ESKAPEE isolates.

Pathogen^*^	**ST type**	**# wastewater isolates^§^**	**Distance to nearest clinical isolate^†^**	**# highly related clinical isolates^‡^**	**AST phenotype**	**Notable AMR genes**
*E. coli* (*n* = 22)	ST-410	2	1	3	MDR	*mcr*-*1.1*, *bla*_CTX − M−55_
		1	51	0	XDR	*bla*_NDM − 1_, *bla*_CTX − M−15_, *bla*_PME − 1_
	ST-744	1	0	2	MDR	bla_CTX − M−55_
	ST-361	1	0	2	MDR	*bla*_NDM − 1_
	ST-131	2	37	0	MDR	*bla*_CTX − M−15_
*K. pneumoniae* (*n* = 10)	ST-147	2	0	10	MDR	*bla*_NMD − 1_, *bla*_OXA − 48_, *bla*_CTX − M−15_, *armA*
	ST-231	2	10	1	MDR	*bla*_CTX − M−15_, *rmtF1*
	ST-37	1	0	1	MDR	*bla*_CTX − M−40_
*P. aeruginosa* (*n* = 9)	ST-233	4	2	1	XDR	*bla*_NDM − 1_, *bla*_PME − 1_, *bla*_VEB − 1_
	ST-235	1	1	16	XDR	*bla*_IMP − 1_, *bla*_NDM − 1_, *bla*_PME − 1_
	ST-654	1	4	11	XDR	*bla*_NDM − 1_
	ST-274	1	2	3	Non MDR/XDR	-
*A. baumannii* (*n* = 5)	ST-2	3	0	>100	XDR	*armA*, *bla*_NDM − 5_, *bla*_OXA − 23_
	ST-164	1	0	6	MDR	*bla*_OXA − 23_, *bla*_OXA − 58_

^*^Total number of wastewater isolates sequenced listed under pathogen name.

^§^Number of wastewater isolates in the same sequence type (ST) as a clinical isolates.

^†^Distance defined as number of allelic differences between wastewater isolate and clinical isolate.

^‡^Highly related clinical isolate defined as clinical isolate with ≤ 10 allelic differences to a wastewater isolate.

For *P. aeruginosa*, we found that 7 of 9 sequenced wastewater isolates shared the same ST type as a clinical isolate for ST233, ST235, ST654, and ST274, all of which showed less than 10 allelic differences to the nearest clinical isolate. Most of these shared strains had the XDR phenotype. Finally, for *A. baumannii*, we found 4 out of 5 sequenced wastewater isolates had the same ST type as clinical isolates for ST2 and ST164, in both cases with no allelic differences. This was most pronounced for ST2, where we had 3 of 5 wastewater isolates and over a 100 clinical isolates that were highly genetically related and had the XDR phenotype.

## 4 Discussion

This study was intended to explore the feasibility of using untreated hospital wastewater to conduct surveillance of MDR pathogens, with a focus on ESKAPEE pathogens. Although there have been numerous studies on AMR wastewater surveillance, few focus broadly across all ESKAPEE pathogens or combine both clinical and wastewater surveillance to assess the clinical significance of wastewater surveillance data (Tiwari et al., 2022, 2024). Here, we carried out parallel clinical and wastewater surveillance at the Queen Sirikit Naval Hospital, in Chonburi, Thailand for a 10-month period from March to December, 2024. We chose to utilize a culture-based approach in order to definitively determine if we could identify ESKAPEE isolates in wastewater that were phenotypically and genetically related to clinical isolates collected at the same site.

ESKAPEE pathogens are common in nosocomial infections, and as such, we hypothesized that untreated hospital wastewater, that contains drainage water from activities such as hand washing and surface cleaning, may contain nosocomial pathogens that are present in the hospital environment. Our results confirmed that untreated wastewater at this hospital was not just rich in drug-resistant bacteria, but that it was specifically rich in ESKAPEE pathogens. ESKAPEE pathogens made up over 50% of isolates collected on selective plates from untreated wastewater. This is in contrast to community water which shows both relatively low load of drug resistant bacteria, and also a very small fraction of drug resistant isolates (2.4%) as ESKAPEE pathogens. We do note that both the overall bacterial load and the percentage of isolates of ESKAPEE pathogens is substantially lower in treated wastewater compared to untreated wastewater—an equivalent of a 20-fold reduction—suggesting that the treatment process kills or disrupts ~95% of ESKAPEE pathogens in the wastewater at this hospital. Our findings are similar to prior studies that found that high rates of drug-resistant ESKAPEE pathogens (Nishiyama et al., 2021; Mutuku et al., 2022; Liu et al., 2023; Kumar et al., 2024) in hospital wastewater, and another that identified ESKAPEE pathogens in untreated hospital wastewater, but not treated wastewater (Irfan et al., 2023).

The most common types of ESKAPEE pathogens that we found in the hospital wastewater were *Enterobacter* spp. (35%) followed by *K. pneumoniae* (29%), and *E. coli* (19%), with *P. aeruginosa* (4%) and *A. baumannii* (4%) making up a small portion of isolates. This is in contrast to the clinical isolates collected which were predominantly *E. coli* (40%), *A. baumannii* (25%), *P. aeruginosa* (15%), and *K. pneumoniae* (15%). This discrepancy could reflect biased selection processes for both clinical isolates and wastewater isolates as well as differences among the pathogens in terms of their ability to disseminate in the hospital environment and to persist in wastewater. We did find that most of the ESKAPEE isolates from untreated wastewater were classified as MDR, with almost all *P. aeruginosa* (91%) and a significant portion of *A. baumannii* (20%) classified as XDR, reflecting the high rate of drug resistance seen among clinical samples at this hospital (Ruekit et al., 2022). Our findings corroborate other studies that have also identified ESKAPEE pathogens in hospital wastewater, albeit with different relative prevalence of the different pathogens suggesting that the profile of ESKAPEE pathogens in hospital wastewater may be highly site-specific or sensitive to the detection approach (Hubeny et al., 2022; Ma et al., 2022; Mustafa et al., 2022; Galarde-Lopez et al., 2024; Krul et al., 2025).

We found that many ESKAPEE isolates contained similar AST profiles as the clinical isolates. With hierarchical clustering, we were able to define distinct AST phenotype clusters and found that the degree of overlap in AST phenotypes between wastewater and clinical isolates varied by pathogen. For *P. aeruginosa* and *A. baumannii*, we found high overlap, for *E. coli* and *K. pneumoniae* we found moderate overlap, while for *E. cloacae*, we found low overlap between wastewater and clinical AST phenotypes. Likewise, we found that most (70 to 100%) of *E. coli, A. baumannii*, and *P. aeruginosa* wastewater isolates shared the same AST phenotype as a clinical isolate suggesting that for these organisms, hospital wastewater isolates have high clinical relevance. By contrast, for *K. pneumoniae* wastewater isolates we found low overlap with clinical AST phenotypes. It is important to note that that sample size plays a significant role in the degree of coverage that wastewater isolates have with clinical isolates. In most AST clusters there was only a single wastewater isolate, suggesting that sample size was a limiting factor in the coverage of clinical isolates. It is possible that by increasing the volume of wastewater collected, we would increase the number of distinct wastewater isolates collected and the coverage of clinical isolates.

Much of AMR surveillance using wastewater sources is focused on AMR gene detection from bulk wastewater samples (Perry et al., 2021; Hubeny et al., 2022; Baba et al., 2023) and we sought to determine if the AMR genes identified in wastewater isolates was comparable to AMR genes found in clinical isolates. We found that for *K. pneumoniae, P. aeruginosa*, and *A. baumannii* PCR-based AMR gene detection of wastewater isolates had moderate sensitivity (60-80%) and high specificity (100%) for identifying clinical AMR genes. For *E. coli*, we found moderate sensitivity and specificity (80 and 60%, respectively), perhaps owing to the high diversity of *E. coli* isolates in both wastewater and clinical samples. For *E. cloacae* we found poor sensitivity and moderate specificity. Overall, these findings suggest that AMR gene detection of bulk samples may be an effective approach for monitoring AMR genes found in clinical ESKAPEE isolates but that further study is needed to validate purely molecular approaches in terms of their clinical relevance.

WGS analysis of A. *baumannii, E. coli, K. pneumoniae*, and *P. aeruginosa* isolates successfully identified wastewater isolates that shared the same ST as clinical isolates that, in many cases were highly genetically related, defined as having ≤ 10 allelic differences by cgMLST. We found that for the more numerous and diverse *E. coli* and *K. pneumoniae* wastewater isolates 15–30% of wastewater isolates were highly related to clinical isolates, while for the rarer and less diverse *P. aeruginosa* and *A. baumannii*, ~80% of wastewater isolates were highly related to clinical isolates. Remarkably, among the 52 wastewater isolates sequenced, we identified two *E. coli* isolates, three *K. pneumoniae* isolates, and four *A. baumannii* isolates that had zero allelic differences to a clinical isolate, indicating that the same clonal lineages that are persisting in patients at the hospital can be found in the untreated wastewater.

Prior studies on direct isolate-level comparisons of clinical and wastewater samples for ESKAPEE pathogens are relatively rare. Culture-based approaches combined with WGS have been successfully used to identify clinical and hospital wastewater isolates and isolates collected from hospital environmental settings, that shared the same ST type and similar AMR gene profiles for *K. pneumoniae* (Constantinides et al., 2020; Krul et al., 2025) and *E. coli* (Constantinides et al., 2020; Davidova-Gerzova et al., 2023). However, despite identifying cases of shared STs between clinical and wastewater or environmental isolates (Constantinides et al., 2020; Baba et al., 2023; Davidova-Gerzova et al., 2023; Krul et al., 2025), few have demonstrated a high level of genetic (Katagiri et al., 2021). In one such example, Katagiri et al. did successfully find a clinical isolate of *E. coli* that showed zero allelic-differences with several isolates collected from hospital sewage (Katagiri et al., 2021).

The present study is notable from these prior studies in several key respects. First, it simultaneously identified a broad range of ESKAPEE pathogens from hospital wastewater. Second, it achieved a high rate of identifying wastewater isolates that share the same ST as clinical isolates (44%, 23 out of 52 wastewater isolates sequenced). Finally, third, a significant portion of the wastewater isolates selected for sequencing (18%, 9 of 50), had zero allelic mismatches with clinical isolates by cgMLST, indicating an extremely high level of genetic-relatedness. The reason we were able to identify wastewater isolates with this level of relatedness to clinical isolates can likely be attributed to three reasons: (1) we used a biased selection process for both wastewater and clinical surveillance to specifically focus on multi-drug resistant isolates, (2) we used AST phenotype clustering to select wastewater isolates with similar phenotypic profiles to clinical isolates, likely enriching for genetically-related wastewater isolates and (3) because we have been conducting clinical AMR surveillance at this hospital for over 7 years, we had a large genomic database of over 1,500 sequences of clinical isolates to compare with our wastewater isolates.

There are some limitations to the present study. First, sampling was a once-monthly single-time point grab sample that may be prone to high variability or otherwise not be representative of the water source. Second, the water volumes collected were based on an initial assessment of bacterial biomass, but subsequent analysis suggested that the sample size of isolates collected was small relative to the diversity of isolates present in the water source, and that larger collection volumes might be necessary. Third, WGS analysis based on a subset of wastewater isolates selected for having shared AST phenotypes with clinical isolates likely enriched for genetically related wastewater isolates and provided a biased indicator of the overall degree of genetic relatedness between clinical and wastewater isolates. Finally, the limited sample size of isolates for WGS in this study (5 to 20 isolates per organism) is too low to provide a reliable estimate of the proportion of wastewater isolates that is highly genetically related to clinical isolates. Further study is needed to characterize the phenotypic and genotypic relationship between wastewater and clinical ESKAPEE isolates.

## 5 Conclusion

In this study we sought to assess the clinical relevance of hospital wastewater surveillance for multi-drug resistance ESKAPEE pathogens in a tertiary hospital in Thailand known to have a high clinical prevalence of nosocomial drug resistant infections (Ruekit et al., 2022). Using a culture-based approach we found that untreated hospital wastewater at this site was rich in MDR ESKAPEE isolates that shared AST phenotypes, AMR genes, and in many cases, high genetic relatedness, to clinical isolates collected at the same site. Notably, in several cases, WGS identified wastewater isolates with zero allelic differences to clinical isolates, suggesting the same strains that are found in clinical cases are being detected in the wastewater. To our knowledge, this is one of the first studies to successfully identify ESKAPEE isolates from hospital wastewater that show high genetic relatedness to clinical isolates across multiple types of ESKAPEE pathogens and underscores the potential for AMR surveillance of untreated hospital wastewater.

## Data Availability

The raw data supporting the conclusions of this article will be made available by the authors, without undue reservation.
