# Supplementary material for: Identification of clinically relevant multi-drug resistant ESKAPEE isolates from hospital wastewater surveillance in Thailand
Source: Front Microbiol. 2025 Sep 10;16:1657219. doi: 10.3389/fmicb.2025.1657219 (PMC12457344; doi:10.3389/fmicb.2025.1657219)
Supplement: Supplementary file 1 [file Data_Sheet_1.docx]

Supplementary Material

##
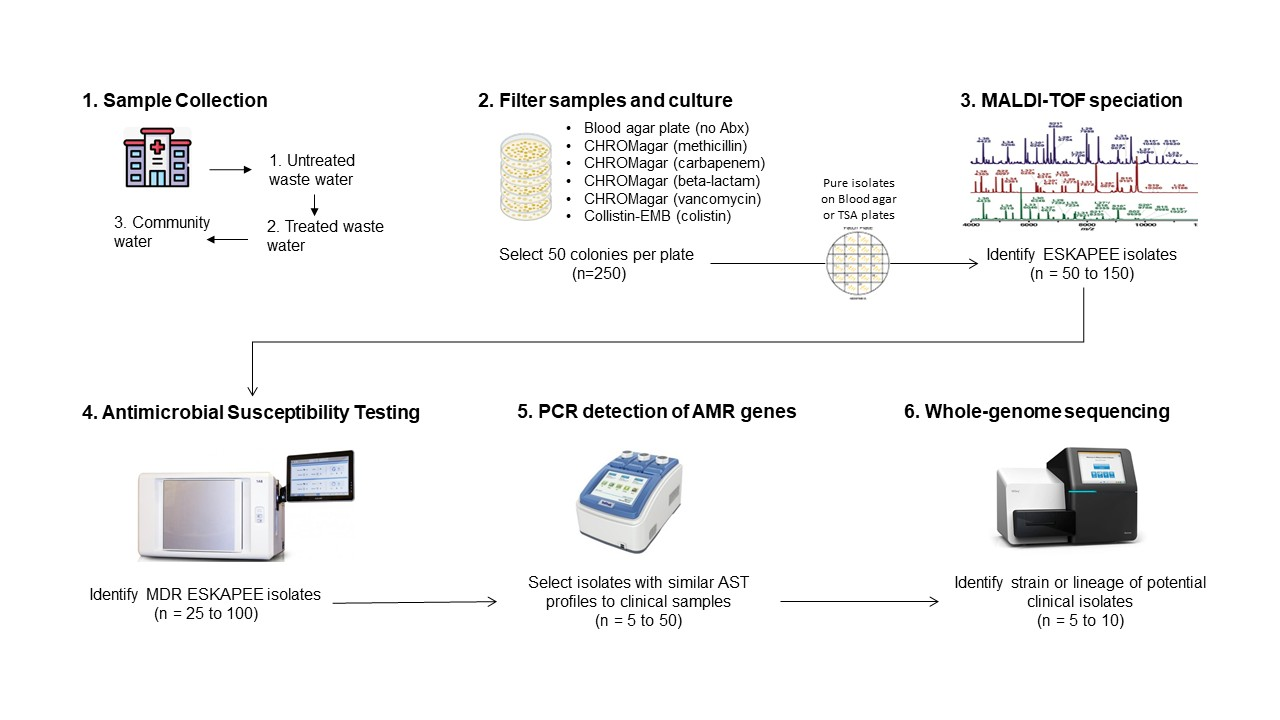


**Supplementary Figure S1.** An overview of the monthly testing process.


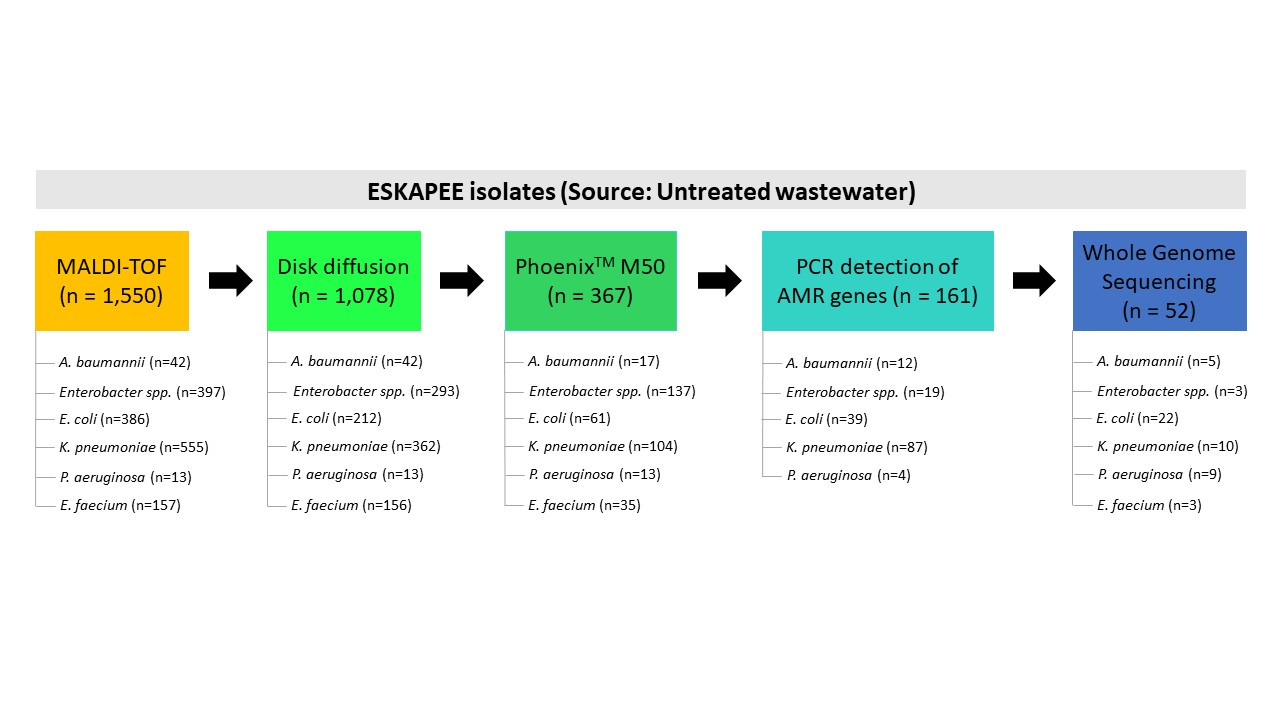


**Supplementary Figure S2.** A flowchart illustrates numbers of ESKAPEE isolates from untreated wastewater characterized by MALDI-TOF through whole genome sequencing analysis.


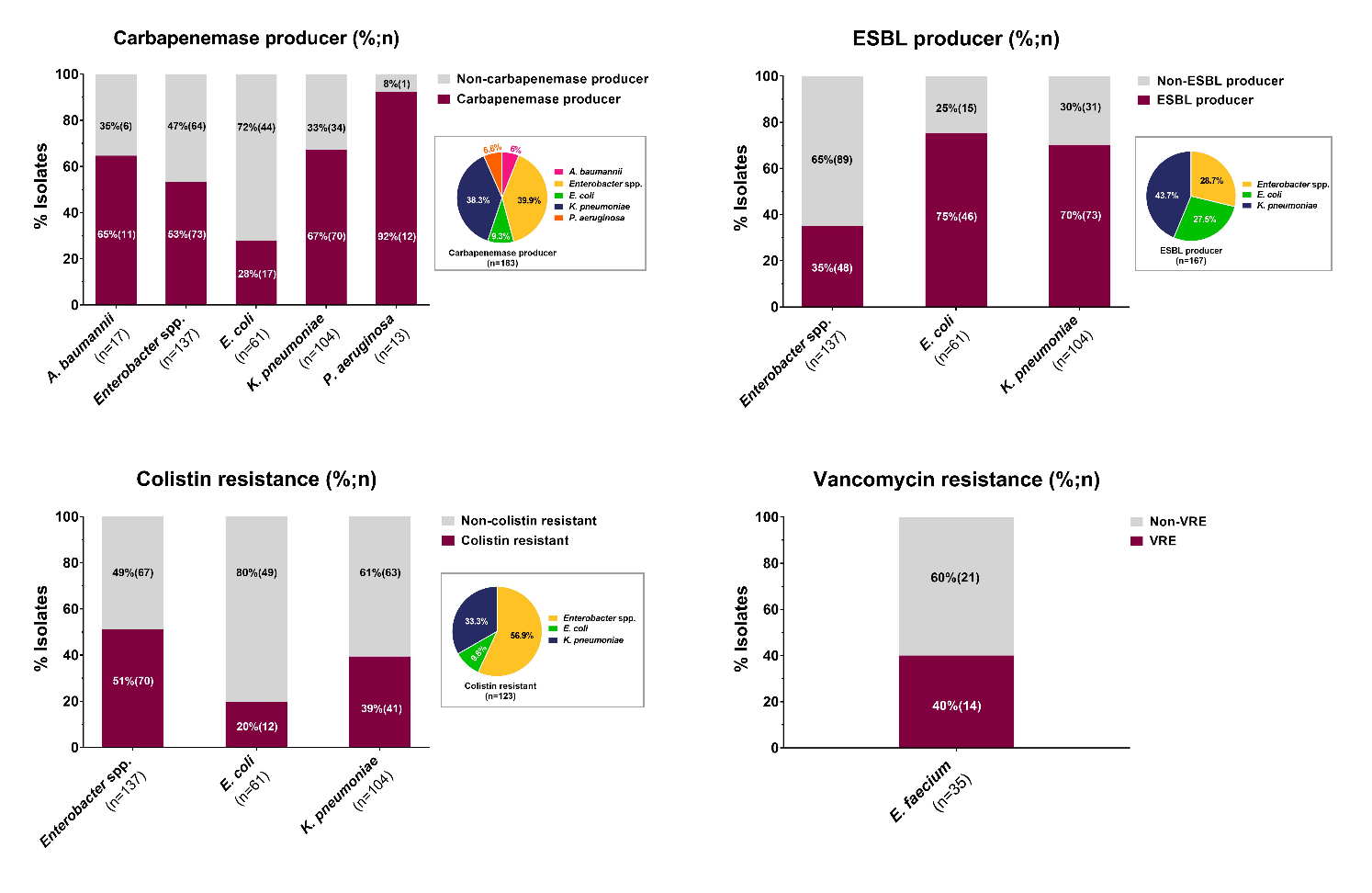


**Supplementary Figure S3.** Production of carbapenemase and ESBL as well as colistin and vancomycin resistance of ESKAPEE isolates from untreated wastewater.


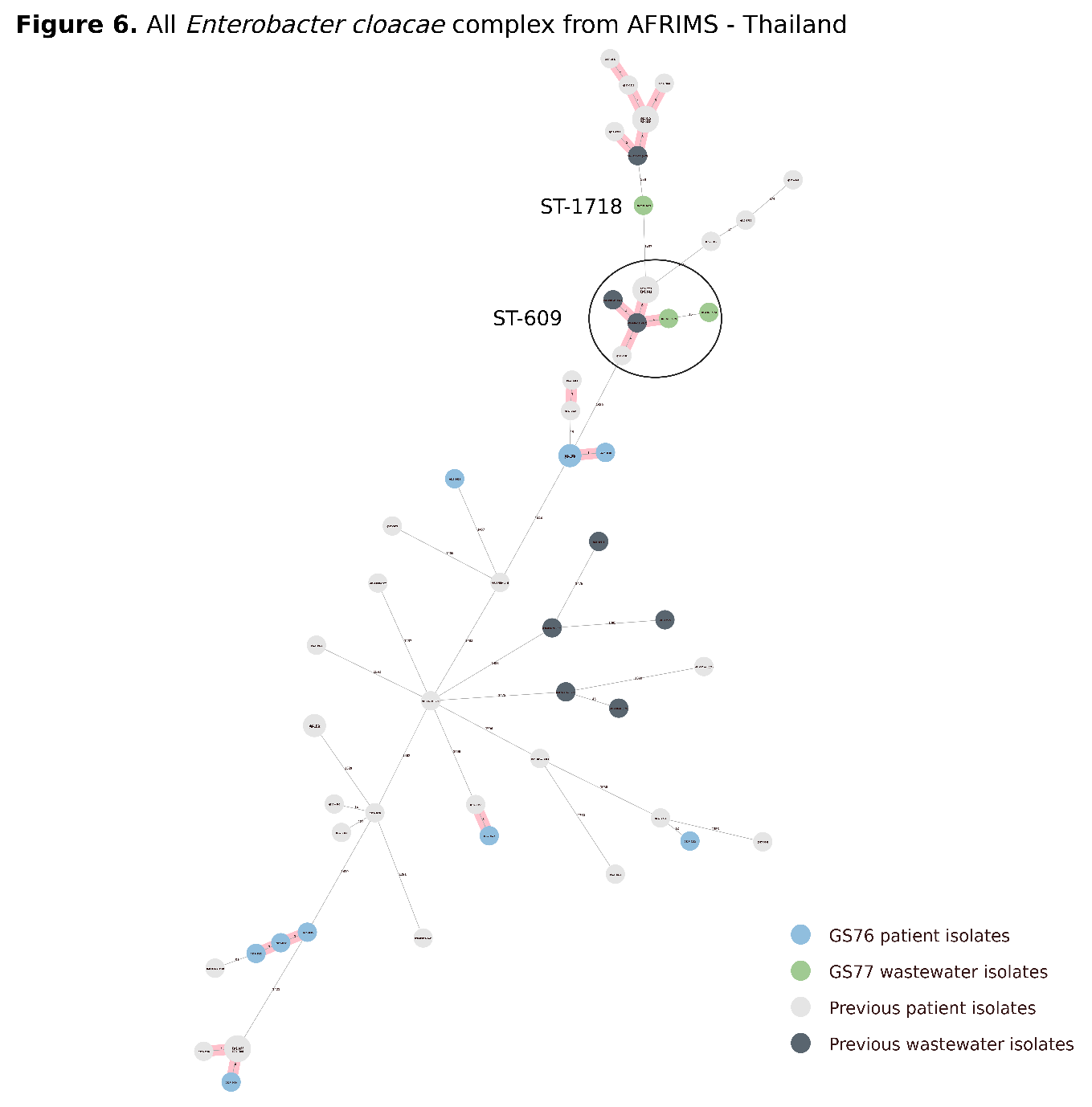


**Supplementary Figure S4.** cgMLST-based minimum spanning trees of *E. cloacae* isolates from untreated wastewater.

**Supplementary Table S1.** Bacterial count from untreated wastewater, treated wastewater, and community water during the collection period of March to December 2024. Six types of agar plate were used for bacterial count assessment.

|  | **Bacterial count on different agar plates (CFU/ml)** | | | | | | | | | | | | | | | | | |
| --- | --- | --- | --- | --- | --- | --- | --- | --- | --- | --- | --- | --- | --- | --- | --- | --- | --- | --- |
|  | **CHROMagar^TM^ mSuperCARBA** | | | **CHROMagar^TM^ MRSA** | | | **CHROMagar^TM^ ESBL** | | | **CHROMagar^TM^ VRE** | | | **Colistin EMB** | | | **Brucella blood** | | |
| **Month of collection (Year 2024)** | **Untreat^1^** | **Treat^2^** | **Com^3^** | **Untreat^1^** | **Treat^2^** | **Com^3^** | **Untreat^1^** | **Treat^2^** | **Com^3^** | **Untreat^1^** | **Treat^2^** | **Com^3^** | **Untreat^1^** | **Treat^2^** | **Com^3^** | **Untreat^1^** | **Treat^2^** | **Com^3^** |
| **March** | 1.48E+05 | 1.00E-01 | 6.12E+03 | 1.13E+05 | 4.62E+02 | 2.25E+03 | 9.53E+04 | 1.00E-01 | 5.40E+03 | 8.55E+04 | 1.00E-01 | 1.41E+04 | Not detected | Not detected | Not detected | 3.10E+05 | 1.35E+03 | 5.14E+04 |
| **April** | 8.18E+05 | 2.97E+04 | 1.38E+03 | 2.95E+05 | 1.57E+03 | 1.01E+03 | 5.58E+05 | 1.28E+03 | 4.02E+02 | 7.28E+05 | 2.01E+04 | 2.01E+03 | Not detected | Not detected | Not detected | 2.47E+06 | 3.21E+04 | 1.38E+04 |
| **May** | 2.15E+06 | 3.46E+06 | 7.82E+03 | 5.88E+05 | 1.97E+05 | 3.49E+03 | 8.76E+05 | 1.05E+06 | 8.84E+03 | 9.15E+05 | 3.62E+06 | 2.49E+04 | 2.39E+05 | 1.00E-01 | 2.39E+04 | 2.32E+06 | 3.12E+08 | 5.06E+04 |
| **June** | 1.54E+06 | 4.99E+04 | 2.88E+03 | 1.11E+05 | 6.58E+03 | 2.46E+03 | 5.44E+05 | 4.20E+03 | 1.94E+03 | 1.18E+06 | 2.11E+04 | 2.93E+04 | 5.90E+05 | 4.37E+04 | 7.58E+04 | 1.49E+06 | 5.02E+04 | 3.89E+04 |
| **July** | 1.25E+06 | 8.23E+04 | 2.92E+03 | 2.08E+05 | 2.28E+04 | 2.80E+03 | 1.34E+06 | 7.60E+03 | 2.35E+03 | 1.61E+06 | 6.56E+04 | 7.52E+03 | 1.52E+06 | 4.75E+04 | 4.79E+03 | 1.11E+07 | 1.97E+05 | 4.94E+04 |
| **August** | 8.89E+05 | 3.46E+04 | 3.67E+03 | 1.07E+06 | 2.29E+04 | 2.97E+03 | 8.55E+05 | 3.23E+04 | 3.51E+03 | 7.15E+05 | 1.63E+04 | 5.99E+03 | 1.86E+06 | 2.08E+04 | 3.27E+03 | 4.53E+06 | 1.31E+05 | 3.25E+04 |
| **September** | 2.37E+05 | 1.89E+03 | 4.14E+03 | 4.41E+05 | 1.86E+03 | 7.10E+03 | 4.26E+05 | 2.66E+03 | 4.86E+03 | 2.13E+05 | 4.14E+03 | 8.91E+03 | 7.93E+05 | 1.65E+04 | 2.36E+03 | 3.71E+06 | 5.18E+05 | 3.93E+04 |
| **October** | 2.11E+05 | 1.55E+03 | 5.54E+03 | 5.14E+05 | 8.70E+02 | 2.61E+04 | 5.54E+05 | 1.23E+03 | 8.69E+03 | 1.92E+05 | 1.47E+03 | 2.54E+04 | 2.84E+05 | 1.02E+04 | 2.94E+04 | 5.31E+06 | 3.28E+04 | 4.77E+05 |
| **November** | 2.19E+05 | 1.55E+04 | 1.77E+03 | 3.68E+05 | 6.08E+03 | 1.22E+03 | 3.54E+05 | 4.68E+03 | 2.84E+03 | 2.21E+05 | 9.54E+03 | 7.11E+03 | 1.84E+05 | 3.26E+03 | 1.13E+04 | 2.76E+06 | 1.19E+05 | 8.18E+04 |
| **December** | 4.92E+05 | 1.59E+04 | 4.14E+03 | 6.74E+05 | 3.50E+03 | 5.10E+02 | 6.63E+05 | 6.44E+03 | 3.87E+03 | 2.77E+05 | 8.64E+03 | 4.73E+03 | 2.50E+05 | 1.39E+04 | 3.44E+03 | 4.63E+06 | 3.17E+05 | 8.59E+04 |

^1^Untreated wastewater; ^2^Treated wastewater; ^3^Community water
